# Spatially Encoded Oncogenesis and Transcriptional Plasticity in Meningioma: Drivers of Therapeutic Resistance and Opportunities for Targeted Intervention

**DOI:** 10.3390/cancers17162694

**Published:** 2025-08-19

**Authors:** Matthew A. Abikenari, Amit Regev, Brandon H. Bergsneider, Vratko Himic, Shreyas Annagiri, Lily H. Kim, Ravi Medikonda, John Choi, Sanjeeva Jeyaretna, Daniel M. Fountain, Michael Lim

**Affiliations:** 1 Department of Neurosurgery, Stanford University School of Medicine, Stanford, CA 94305, USA; mattabi@stanford.edu (M.A.A.); amitre@stanford.edu (A.R.); bbergs@stanford.edu (B.H.B.); sa27@stanford.edu (S.A.); lilyhkim@stanford.edu (L.H.K.); rmediko1@stanford.edu (R.M.); johnhchoi@stanford.edu (J.C.); 2 Department of Neurological Surgery, University of Miami Miller School of Medicine, Miami, FL 33136, USA; vxh268@med.miami.edu; 3 Department of Neurological Surgery, Oxford University Hospitals, University of Oxford, Oxford OX1 2JD, UK; sanjeeva.jeyaretna@ouh.nhs.uk; 4 MRC Weatherall Institute of Molecular Medicine, University of Oxford, Oxford OX1 2JD, UK

**Keywords:** meningioma, molecular profiling, embryogenesis, tumor grading, transcriptomics, targeted therapies

## Abstract

Whilst typically benign, a subset of meningiomas displays aggressive and recurrent behavior. There is a paucity of reliable treatment options for this subset of patients and a relative lack of consensus on how to best manage these patients. This clinical challenge reflects underlying molecular complexity, driven by *NF2*, *TRAF7*, and *CDKN2A/B* mutations alongside pervasive epigenetic dysregulation. High-throughput molecular profiling studies have proposed biologically distinct meningioma subgroups with varying clinical trajectories and therapeutic vulnerabilities. Distinct cell lineages of meningeal precursors are now appreciated to be essential in the establishment of the meninges. The numerous cellular lineages involved in meningeal development, the heterogeneity of meningioma location and (epi)genomic behavior, and the variability in its clinical and radiological manifestations raise the question of what critical insights can be gained by understanding meningeal development during embryogenesis to understand meningioma tumorigenicity. The current paper examines this paradigm by highlighting spatially linked mechanisms of anaplasia and treatment resistance, including the role of neural crest-derived convexity meninges in promoting dedifferentiation via YAP/TAZ signaling and mesoderm-derived skull base regions in maintaining *TRAF7*-mediated vulnerabilities. We further elucidate the emerging synthetic lethal paradigms, CRISPR-enabled target discovery, and PROTAC-mediated degradation strategies that may transform the therapeutic landscape of clinically challenging meningiomas driven by complex oncogenic circuitry. By bridging embryogenesis, spatial genomics, and molecular targeting, we propose a developmentally informed, lineage-stratified model for advancing precision therapeutics in high-grade and recurrent meningiomas.

## Simple Summary

Meningiomas are common brain tumors, but a subset behaves aggressively and resists current treatments. This review proposes a developmentally informed framework showing that where a meningioma arises in the skull mirrors its embryologic origin (neural crest vs. mesoderm) and imprints distinct genetic, epigenetic, and immune programs that shape tumor behavior and treatment response. This lineage “stamp” helps explain why convexity and skull-base tumors differ and highlights actionable circuits, NF2/YAP–TAZ, PI3K/AKT, TRAF7-linked pathways. We integrate spatial genomics and single-cell data to map tumor location and molecular class to rational therapies, including CDK4/6, PI3K/AKT, YAP/TEAD, and epigenetic inhibitors, with emerging strategies such as synthetic-lethal combinations, CRISPR-guided target discovery, and degrader (PROTAC) approaches for high-grade and recurrent disease. Our goal is a practical roadmap that links anatomy and biology to precision treatment and trial design, improving outcomes by matching the right therapy to the right meningioma at the right site.

## 1. Introduction

Meningiomas are the most common primary intracranial tumors of the central nervous system, comprising approximately one-third of all CNS neoplasms and some of the most prevalent extra-axial tumor diagnoses seen in the practice of neurosurgery [1,2,3]. Many are asymptomatic; however, progressive tumor expansion can result in focal compression of surrounding neural tissue and mass effect, manifesting with headache, generalized seizures, altered mental status, diplopia, sensorimotor deficits, progressive spastic weakness, and other symptoms based on tumor location [2,4].

Meningiomas are grouped by histological grading and show wide histological heterogeneity with overlapping characteristics between mesenchymal and epithelial cell types [5,6]. Meningioma annual incidence is 6 per 100,000 population, with the incidence rate being highest after the fifth decade of life. Meningiomas are twice as common in women compared to men [5,6]. While the majority of meningioma cases are benign (WHO Grade I), some of the subtypes show malignant behavior (WHO Grade II and III) [7]. Meningiomas are thought to arise from arachnoid cap cells, though Kalamarides et al. suggest they may originate from PGDS-positive progenitor cells, including arachnoid barrier and dural border cells [8].

To date, there are no effective targeted therapies for patients with aggressive malignant meningiomas. Our poor understanding of meningioma biology and tumorigenesis has hampered many novel treatments [9]. Established risk factors for meningioma, genetic signatures, disease-altering phenotypes, and potential therapeutic vulnerabilities need further interrogation [7,10,11]. Although malignant meningiomas’ exact etiology and oncogenesis remain inconclusive, substantial progress over the last two decades has deepened our understanding of genotypic features, phenotypic drivers, and therapeutic vulnerabilities [2,12].

One way to better understand meningioma tumorigenesis is to examine the development of the meninges. Until recently, the meningeal layers were viewed merely as specialized membranes surrounding brain tissue, serving a purely protective role [13]. However, increasing evidence suggests otherwise. Meninges are not mere barriers; they orchestrate critical phases of development, provide essential contributions to neurogenesis, and influence the neuroimmunological environment [13,14,15]. Additionally, the meninges are critical in developing the blood–brain barrier (BBB), an essential component of the relative immune privilege of the brain [16]. Recent data suggest that the meninges directly affect brain and calvarial bone development [14,15,17,18,19]. Dysfunctions in the meninges have been linked to cranial deformation and neuro-developmental disorders, including Dandy-Walker malformations and cobblestone lissencephaly [13,15]. Meningeal development begins in the first trimester, when the meningeal layers are highly responsive to environmental stimuli and genetic control, making them most vulnerable to developmental disruptions and epigenetic alterations that can result in structural malformations and future tumor susceptibility [18].

Along with genetic and epigenetic determinants, the immune contexture of meningiomas is also increasingly seen as a critical driver of tumorigenesis and aggressiveness. High-grade meningiomas are also significant in sharing features with glioblastoma, including an immune-excluded, metabolically reprogrammed TME dominated by tolerogenic macrophages and FOXP3^+^ regulatory T cells, with low tumor mutational burden and impaired antigen presentation [20,21,22,23,24]. Furthermore, immunotherapy efforts are beginning to leverage these vulnerabilities; notably, mesothelin was identified as a surface antigen on human meningiomas, and CAR T cells targeted against mesothelin had robust cytotoxicity and durable regression of tumor in patient-derived organotypic cultures and orthotopic xenografts, providing the first preclinical rationale for CAR T therapy in treatment-refractory meningiomas [25]. In addition, the immunotherapeutic potential of CAR-T cells is increasingly elucidated in the emergent literature across a spectrum of malignant CNS neoplasms [26,27]. These findings define the intersection of immune modulation and metabolic orchestration in meningioma biology and emphasize the need to include immunotherapeutic interventions within the molecularly guided translational paradigms of the future [28].

The spatial phenotypes defined in meningiomas have also provided evidence that distinct cell lineages may drive distinct tumor subtypes. In the current review, we summarize evidence spanning pivotal mid-20th-century preclinical embryological studies through contemporary epigenomic analyses supporting the role of the meninges as a critical site of meningioma tumorigenesis. We provide a review of current meningioma diagnosis, histological grading, presentation, and treatment options. We then summarize the pathophysiology of meningiomas, meningeal embryology, and potential biological drivers for future therapies.

## 2. Foundations of Clinical and Molecular Pathogenesis in Meningioma

Traditionally diagnosed through neuroimaging and histopathology, meningiomas have been graded by the World Health Organization (WHO) into three grades based on mitotic activity and cell features. Grade I tumors are indolent and represent the majority of cases. In contrast, Grades II and III are characterized by heightened mitotic activity, invasion into the brain, and, in Grade III, are malignant [29,30]. Morphologic criteria, nevertheless, have been insufficient by themselves for the prediction of behavior or the planning of therapy [11]. Indeed, although classically defined as benign, certain grade I meningiomas may display phenotypes of pronounced aggression, invasion into peripheral tissue, and resistance to therapeutics, particularly upon recurrence. Efforts to discriminate between traditionally indolent grade I meningiomas and the more clinically aggressive, “grade 1.5” phenotype for improved prognostic value have relied on the identification of distinctive molecular markers [31,32]. Updated 2021 WHO CNS5 classification and cIMPACT-NOW guidelines now incorporate molecular markers, such as *CDKN2A/B* deletions and *TERT* promoter mutations [33], that precede histologic appearance to upstage tumors [34,35]. However, histologic uncertainty and radiographic overlap, particularly in asymptomatic or incidentally discovered cases, still highlight the clinical necessity for integrated molecular diagnostics [36].

Heterogeneity is also highlighted by the variation in clinical presentation. Meningiomas are often symptom-free or have focal neurological deficits secondary to mass effect, edema, or involvement of eloquent structures [35,37]. While resection remains the principal treatment, gross total resection is commonly limited by proximity to neurovascular structures [38,39]. Radiation therapy is an adjunct in inoperable or recurrent tumors, and systemic treatments, primarily investigational, have been of limited value. Despite the growing use of targeted treatments directed against hormonal, angiogenic, and proliferative pathways, no disease-modifying therapy is yet standard of care [40,41,42,43]. This therapeutic plateau reflects the greater quandary: conventional approaches fail to consider the inherent genomic and developmental complexity of these tumors.

Pathogenesis of meningioma is controlled by disparate mechanisms at the molecular level, such as chromosomal instability, cell cycle deregulation, angiogenesis, and inflammation. The most well-characterized genomic change is biallelic inactivation of the 22q *NF2* gene in nearly half of all tumors, with a high frequency in convexity meningiomas. *NF2* loss deregulates Hippo signaling, permitting unrestrained YAP/TAZ activity and oncogenic growth [44,45]. Grade II/III meningiomas also feature additional genomic complexity, losses in chromosomes 1p, 9p, 10, 14, and 18, along with dysregulation of the pRB and p53 tumor suppressor pathways [1,45,46]. Aberrant expression of angiogenic factors like VEGF-A also facilitates peritumoral edema, recurrence, and progression [47,48].

**Figure 1:**
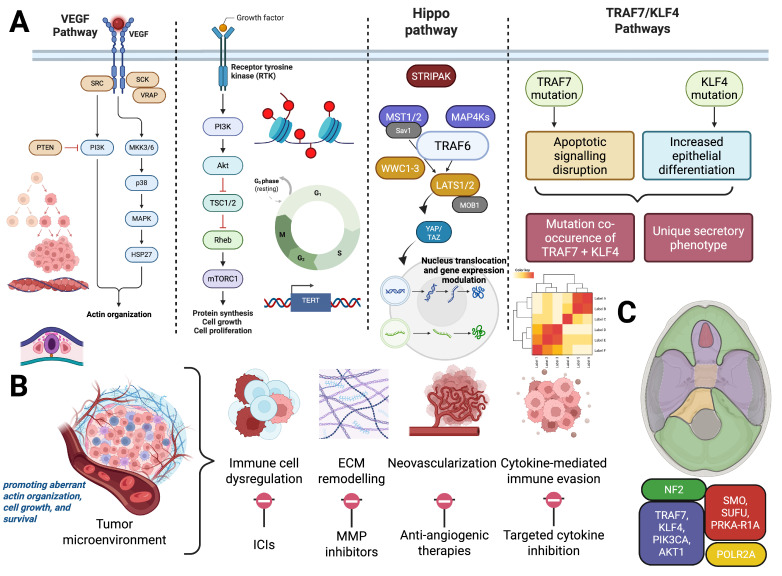
Integrated Molecular, Spatial, and Microenvironmental Framework of Meningioma Pathogenesis and Therapeutic Vulnerabilities. (**A**) Key molecular alterations implicated in meningioma tumorigenesis. Left: Dysregulation of VEGF/MAPK and PI3K/mTOR pathways driven by upstream mutations in NF2 or TRAF7-related gene networks as well as KLF4 mutation pathways, aiding in increased epithelial differentiation, promoting aberrant actin organization, cell growth, and survival. Right: Epigenetic dysregulation (histone modifications), telomerase reactivation via TERT promoter mutations, and CDKN2A/B deletions contribute to cell cycle progression and immortalization. (**B**) Hallmarks of the meningioma tumor microenvironment and associated therapeutic targets. These include immune cell dysregulation (targeted via immune checkpoint inhibitors), ECM remodeling (addressed with MMP inhibitors), angiogenesis (treated with anti-angiogenic therapies), and cytokine-mediated immune evasion (targetable through cytokine blockade). (**C**) Spatial distribution of driver mutations in meningiomas mapped across embryologically distinct meningeal regions. NF2 alterations predominate in the posterior and lateral skull base (green), while mutations in TRAF7, KLF4, AKT1, and POLR2A localize more anteriorly (red and yellow), reflecting embryologic origins and site-specific tumor biology. Created with BioRender.com.

Figure 1 recapitulates the molecular, spatial, and microenvironmental framework of meningioma pathogenesis and therapeutic vulnerabilities.

Efforts now have shifted towards molecular subclassification and precision targeting. Trials such as NCT02523014 now stratify patients based on *SMO*, *AKT1*, and *CDK4/6* mutations, to individualize therapy to the tumor’s oncogenic circuitry [47,48,49,50]. Simultaneously, evidence accumulates for the role of inflammatory mediators, especially COX-2 and prostaglandins, in enhancing tumor growth and immune evasion, with a preference in high-grade meningiomas [50,51,52]. Unlike gliomas, which have been the focus of extensive immunophenotypic and transcriptomic subclassification, molecular classification of meningiomas has historically lagged behind [52,53,54], Emerging genomic subgroups, including *NF2*-inactivated tumors, 1p/22q co-deleted lesions, and non-*NF2* mutant subsets such as those harboring *AKT1*, *SMO*, *PIK3CA*, or *KLF4* mutations, are reshaping treatment paradigms by integrating biological behavior into clinical decision-making [47,48,49,50]. Table 1 summarizes the key genetic and epigenetic alterations in Meningiomas.

Cumulatively, these developments represent a paradigm shift: away from static histologic grade and toward dynamic, molecularly informed risk stratification. The remainder of the current review expands upon this revolution by investigating the embryologic and spatial underpinnings of meningioma formation with the hypothesis that developmental biology, previously overlooked in favor of descriptive pathology, holds the key to the heterogeneity that has long stalled clinical progress.

## 3. The Role of the Meninges in Meningioma Tumorigenesis

### 3.1. Genotypic Features and Spatial Phenotypes of the Meninges

Numerous large-scale genetic studies, as well as preclinical meningioma modeling, have produced a transparent and replicable relationship between meningioma embryology and tumorigenesis, in particular between the anatomical location of meningioma and distinct pathogenic variants driving tumorigenesis [43,51,55]. Utilization of molecular marking for neural crest cells, conditional knockout mice studies, and single-cell transcriptomics have yielded substantial progress in the characterization of meningeal embryogenesis and the spatial phenotypes of genetically distinct meningiomas [43]. In particular, such emergent models have demonstrated that skull-based meningiomas are derived from mesodermal tissue, while convexity-based meningiomas are derived from ectodermal tissue neural crest cells [43,55,56].

Transcriptomic approaches such as single-cell RNA sequencing analyses of meningeal fibroblast in the prosencephalon have demonstrated transcriptionally distinct fibroblast populations across different brain regions [43,56,57]. The corresponding studies revealed that neural crest cell lines give rise to the anterior meninges. In contrast, mesodermal tissue gives rise to the posterior meninges, revealing the presence of regionalization in gene expression. Numerous other studies have also demonstrated a relationship between distinct pathogenic variants of the genome and the locational characteristics of tumorigenicity in meningiomas [56,57,58]. In particular, molecular profiling of 86 sequenced samples of meningiomas with distinct spatial phenotypes has revealed the role of neural crest gene expression as a major driver in meningioma tumorigenesis, indicating that meningioma tumorigenesis relies on gene regulatory networks and subsequent misactivation and dysregulation of developmental cell population trajectories [59,60,61]. Although there are numerous reported pathogenic variants driving tumorigenicity and the distinctly presented spatial phenotypes in meningioma patients, below we focus on the role of neurofibromatosis type 2 (*NF2*) and TNF receptor-associated factor 7 (*TRAF7*) mutations driving tumorigenicity across convexity and skull-base meningiomas, respectively.

Skull base (SB) meningiomas represent nearly half of all surgical meningioma cases. They are often challenging to resect entirely due to their proximity to the cranial nerves and critical cerebral vasculature [60,62,63]. Given the need for improved treatment strategies, elucidation of the underlying pathological mechanisms of SB meningiomas carries enormous therapeutic significance. One particularly striking effort in interrogating the underlying pathological genetic variants in SB meningioma has been the implication of *TRAF7* dysfunction [62,63]. Multiple next-generation sequencing technologies studies have identified somatic *TRAF7* driver mutations in anterior SB meningiomas. In particular, the pathological and phenotypic diversity associated with *TRAF7* mutations driving tumorigenicity can be partly explained through embryologic origins [59,60,63]. For instance, meningiomas harboring *TRAF7* mutations are nearly always localized ventral to the foramen magnum of the skull base, a region now recognized as being derived from mesodermal meninges, consistent with the spatial-genotypic patterns linking *TRAF7*-driven tumorigenesis to mesodermal embryologic origin [63,64,65].

Convexity meningiomas account for approximately 20% of all meningioma cases and, unlike skull base counterparts, are frequently amenable to gross total resection. Their tumorigenesis, however, is shaped by distinct molecular drivers that underlie their unique anatomical distribution and spatial phenotype [40,65,66]. The most predominant variant persistently present in convexity meningiomas is the *NF2* mutation. As previously mentioned, mutations in *NF2*, the first ever identified driver of genetic pathogenesis in meningiomas, are responsible for the regulation and production of the key tumor suppressor protein Merlin which inhibits PI3K intracellular signaling pathways and activates the mammalian hippo signaling pathways. Two pieces of evidence place *NF2* mutations at the center of pathogenic drivers in convexity meningiomas. First, roughly 50–75% of *NF2* patients develop convexity meningiomas; second, approximately 60% of sporadic convexity meningiomas present with pathogenic variants of *NF2*. Functionally, *NF2* loss promotes tumorigenesis by disrupting the Hippo pathway, leading to aberrant nuclear translocation of YAP/TAZ and resultant activation of proliferative and anti-apoptotic gene programs. *NF2* is also crucial for contact inhibition and adherens junction integrity by E-cadherin; its loss removes cytoskeletal structure and cell–cell adhesion, further enhancing oncogenic advancement [62,63].

More recent work from our lab has added another layer to the molecular landscape by describing a hormonally controlled PI3KCA–PI3K–AKT pathway in a subgroup of meningiomas, involving endocrine signaling in tumorigenesis through isoform-specific PI3KCA mutations [67]. These findings augment the established *NF2*–Merlin and Hippo pathway alterations in convexity meningiomas by describing an alternative oncogenic pathway with enrichment in skull base and non-*NF2* tumors with a significant predilection for progesterone receptor-positive tumors. Mechanistically, PI3KCA activation converges on AKT–mTOR signaling while sculpting the immune-metabolic milieu, favoring increased Treg recruitment and impaired CD8^+^ effector function, features characteristic of the tolerogenic tumor microenvironment of glioblastoma [68,69,70]. Importantly, our work identifies the therapeutic potential to target this axis using PI3K isoform inhibitors and endocrine manipulation, bridging molecular profiling, immune reprogramming, and metabolic regulation into a precision framework for a biologically distinct meningioma subtype [67].

Meningiomas are also classified into one of six methylation classes: MC benign 1, 2 and 3, MC intermediate A and B, and MC malignant. Previous work has demonstrated the potential use of these methylation classes for improved prognostic ability and prediction of recurrence [71,72,73,74]. It is critical to note, however, that while the meninges demonstrate distinct methylation classes by region during development, there is mixed evidence as to whether the methylation classes of developing meninges and meningiomas are associated.

### 3.2. Embryogenesis of Brain Meninges

Understanding the meninges through the developmental context can provide significant insights into the cellular and molecular mechanisms driving meningioma tumorigenesis. Exploring their embryogenesis offers a contextual foundation for unraveling their biological complexity, which leads to tumor development and progression.

The establishment of the meninges starts during the 4th gestation week (GW) (Carnegie Stage 13 and embryonic day ~(E)8.5 in mice) with the encapsulation of mesenchymal cells around the hindbrain at the time of neural tube closure [13,18]. Subsequently, the mesenchymal cells distribute to the midbrain and forebrain—forming a mesenchymal sheath by the end of 5th GW (Carnegie Stage 15, (E)9.5) [13,75]. This sheath is the primordium of the meninges, skull, and scalp [13]. Concurrently, fibroblast cells within the developing pia layer produce extracellular matrix proteins (i.e., collagen IV, proteoglycans) to form the pial basement membrane, separating the meninges from the rest of the brain. By stage E10.5, the established mesenchyme sheath (primary meninx) is composed of two clearly defined layers: the “outer layer” and the “inner layer” [13].

By Carnegie 17 (6th GW and E13), the outer layer will develop into the dermal layer and the calvarial layer (which gives rise to the skull and cranial sutures). Simultaneously, the inner layer from stage E10.5 will give rise to two distinct layers: the pachymeninx and leptomeninx. Subsequently, the pachymeninx (dura mater) and leptomeninx (arachnoid and pia mater) differentiate in basal to apical direction, forming the three meningeal layers [13,76].

The intricate differences between the meningeal layers go beyond their structural order surrounding the brain. The anatomical distribution of the three layers is predetermined by cell lineage groups that were recently discovered [43]. To contextualize the role of the meninges in brain tissue development, we will delve deeper into their cellular origins.

### 3.3. The Cellular Origin of the Meninges

In recent years, the availability of genetic and molecular markers for long-term lineage tracing has revealed that all meninges arise from mesenchymal progenitors. However, such progenitors do not stem from a common shared population but instead arise from alternative embryonic sources, such as mesodermal and neuroectodermal (neural crest-derived) mesenchymal lineages. Primitive embryonic layers contributing to the developing mesenchyme include the mesoderm and neural crest cells [43,77,78,79].

Early research conducted three decades ago using quail-chick chimeras and species-specific nuclear staining techniques revealed that the meninges overlying the forebrain are predominantly of neural crest origin, whereas those over the brainstem are derived from mesodermal progenitors [78,80,81]. Subsequent work employing HNK1 expression in spinal meninges demonstrated that, at the spinal level, the pia mater arises from neural crest cells while the dura and arachnoid mater are mesodermal in origin [43,79]. These findings indicate that meningeal embryonic derivation differs both by brain region and by meningeal layer.

Recent lineage-tracing studies in transgenic mice using molecular markers such as *Wnt1-Cre* and *Sox10-Cre* (for neural crest), *Mesp1-Cre* (for mesoderm), and *PGDS-Cre* (for arachnoid and dural border cells) have refined our understanding of regional meningeal ontogeny [77,82,83]. These studies show that the meninges overlying the cerebral hemispheres (including hemispheric dura) are largely neural crest-derived, while those overlying the midbrain, hindbrain, and ventral brainstem originate from mesoderm [43,82]. While these lineage-tracing data strongly support a predominantly neural crest origin for the meninges overlying the cerebral hemispheres, including the hemispheric dura, some studies suggest regional heterogeneity and potential mesodermal contribution within the deep convexities. This reflects an emerging view that the hemispheric dura may represent a developmental interface zone, where neural crest–derived cells predominate but are not entirely exclusive, highlighting the complex spatial patterning of meningeal ontogeny. Importantly, although the cerebellum lies within the posterior cranial fossa, the meninges overlying the dorsal cerebellar convexities are neural crest-derived, whereas those at the ventral skull base and foramen magnum, including the dura, are of mesodermal origin [18,19,43,84].

Finally, bulk single-cell analyses using Cre-Lox-based fluorescent lineage tracing have further emphasized that meningeal origin diverges along the anterior–posterior axis. These studies found that anterior cranial meninges are enriched in neural crest derivatives, while posterior and ventral cranial meninges, particularly near the skull base, are primarily mesodermal in origin [76,85,86].

The latest developments in understanding the diverse cellular origins of the meninges have significant clinical implications. For example, it may explain why certain brain regions are more prone to meningioma and tumor development [43,87]. Convexity meningiomas often develop from the dura mater [88]. In contrast, SB meningiomas develop from the arachnoid layer of the meninges [3,43]. The dura mater and the arachnoid mater are derived from the mesoderm, indicating a potential cellular vulnerability with a common origin. Namely, early aberrant cellular events within the mesoderm lead to tumorigenesis and skull-based impairments [19,89]. Indeed, other congenital skull malformations, such as craniosynostosis, have been associated with impaired maturation of the meninges due to aberrant gene expression [90]. In this way, the meninges and the overlying calvarium development are closely interlinked, develop in concert, and should be discussed in this way.

### 3.4. The Meninges Orchestrates Calvarial Development

The proximity of the meninges to the calvarial bone anticipates a developmental link between them [13]. This topic was initially studied in 1979 when the calvarial bone was surgically removed or injured in fetal, neonatal, and young postnatal rats and rabbits [91,92,93]. In these experiments, either through removal or transplantation, the dura mater was deemed indispensable and sufficient for the re-ossification of the calvarial bone [91,93].

Recently, the role of the meninges in calvarial development has been restated. Based on evidence from the E10.5 mouse embryo, the calvarial bone comprises five regions: two frontal, two parietal, and one interparietal bone, all connected by soft sutures [13]. Similarly to the development of the meninges, the calvarial bone develops from the mesenchymal cells surrounding the brain. As such, the progenitor cells of the calvarial bone are determined as part of the previously mentioned primary meninx. Notably, the frontal bone of the calvaria is mainly derived from neural crest cells (with minor mesodermal contribution), whereas the parietal bone is exclusively mesodermal. The interparietal bone contains both mesoderm and neural crest cells [13].

Towards the final stage of development, the meninges and the calvarial bone exhibit a significant degree of continuity—the dura’s outer layer serves as the periosteum of the inner calvarial bone [13]. In Carnegie stage 17 (6 GW and ~E13), the meningeal layers differentiate towards the apex as the frontal and parietal calvarial bones arise from the mesenchyme on the basolateral side of the cranium [93,94]. Additionally, the expansion of the dural limiting layer is significantly correlated with the growth of the calvarium. The dura is involved in calvarial bone development, likely by providing osteogenic signals that control calvarial morphogenesis [13]. Moving forward, we explore the regulatory signals between the meninges and the other developing cranial structures, including the calvarian bone and the cerebral cortex, demonstrating shared meningeal signaling pathways that synchronize osteogenesis with cortical neurogenesis.

### 3.5. Genes and Signaling Factors of the Meninges Regulate Cerebral and Calvarial Development

#### 3.5.1. Meningeal Derived Molecular Signals Governing Cortical Neurogenesis and Neuronal Migration

In 1999, researchers reported that when the neural crest was removed in mice, the forebrain neuroepithelium underwent apoptosis and ultimately degenerated [95]. However, it was unclear which meningeal signaling factors led to such a decline in cellular viability. While little is known about meningeal gene signaling and the regulation of neighboring cerebral structures, significant efforts have been made to answer this vital question in recent years.

Evidence from mice studies showed that the meninges release trophic factors that determine the positioning and maturation of neurons around the brain tissue [15,17,96,97]. Remarkably, the dura and subarachnoid mater secrete CXCL12 (chemokine (C-X-C motif) ligand 12, also known as SDF-1) by the activation of its associated FOXC1 transcription factor (TF) [98]. When the ligand CXCL12 arrives in neuronal cells that express its corresponding receptors (CXCR4 and CXCR7), it leads to the maturation and migration of those neurons [17,98]. Accordingly, in a further murine study testing the loss of transcription factor (TF) Foxc1 (also known as Mf1), the forebrain meningeal formation was significantly impaired [15].

The expression of Foxc2 has also shown critical involvement in cranial regulation, as mice with mutant Foxc2 exhibited a significant reduction in osteoprogenitor cell maturation [99]. Intriguingly, in murine studies of reduced zinc-finger transcription factors called Zic1 and Zic3, forebrain development was compromised, and the expression of Foxc1 and Cxcl12 was significantly downregulated [100].

Other important meningeal-brain factors have been identified, such as Retinoic Acid Receptor Alpha (RARA) transcription factor. In mice models with RARA-gene deletion, neuronal migration was altered, and the cerebral forebrain and hindbrain layers were substantially disorganized [101]. In mice studies that ablated the meninges, when RARA alone was introduced, it was sufficient for neurogenesis and the rescue of cortical defects associated with Foxc1 [15].

The meninges also secrete signaling molecules that can repel neuronal maturation and migration [13]. For instance, the regulatory proteins of genes BMP4, BMP7, and TGFβ1 are secreted from the meninges into the ventral forebrain and repel oligodendrocyte precursor cells into the cerebral cortex during mouse embryogenesis [13,102]. Further, the meninges were shown to affect the corpus callosum formation; when BMP7 was produced, it inhibited axonal callosal outgrowth [102].

Importantly, primary genomic or structural defects in the meninges were also shown to cause disruptions of the pial basement membrane (BM) [42]. The pial BM is a physical barrier and is imperative for guiding neuronal migration and proper organization of the cerebral cortex and the cerebellum [103]. Mice with focal chemical ablation of the meninges experienced pial-BM breakdown. When meningeal cells were introduced back at the damaged site, pial-BM structure and function were recovered [104].

In recent years, defects in the meninges have been linked to neurodevelopmental diseases through the discovery of related genetic mutations and meningeal abnormalities. For example, human imaging studies in individuals with cobblestone lissencephaly (CL) showed that the cerebral cortex was smooth and abnormally thickened instead of ridges and grooves [105,106]. This phenotype is mainly expected when the pial-BM is compromised, leading to excessive migration of neurons into the meninges [13]. Additionally, Dandy-Walker malformation is another neurodevelopmental disease characterized by the accumulation of CSF inside the brain and cerebellar hypoplasia, which has been closely linked to *FOXC1* gene mutations [105].

#### 3.5.2. Convergence of Osteogenic and Neurogenic Pathways in Calvarian Development

Beyond the cerebral cortex, researchers have further indicated that the meninges secrete signaling molecules that regulate the role of calvarial bone development. Various animal studies from the past two decades have reported that dural signals, such as TGFβ, FGF (fibroblast growth factor), and those from the BMP (bone morphogenetic protein) family mediate calvarial re-ossification after injury [13,92,107,108,109].

Gene regulation’s diverse and critical role in calvarial and cerebral morphogenesis is still preliminary but promising and thus requires further attention. Over the past two decades, the aforementioned signaling factors have also been shown to play an essential role in mesenchyme development [110]. However, the differential expression between the mesenchyme derivatives (neural crest and mesoderm cells) was versatile and substantially different from one another [110,111]. For example, the Wnt signaling pathway and *FOXG1* gene play a critical role in the induction and migration of neural crest cells [110,111,112]. In mesodermal cells; however, the genes *BMP* and *FGF* were crucial for the proper maturation of the meninges [113].

**Figure 2:**
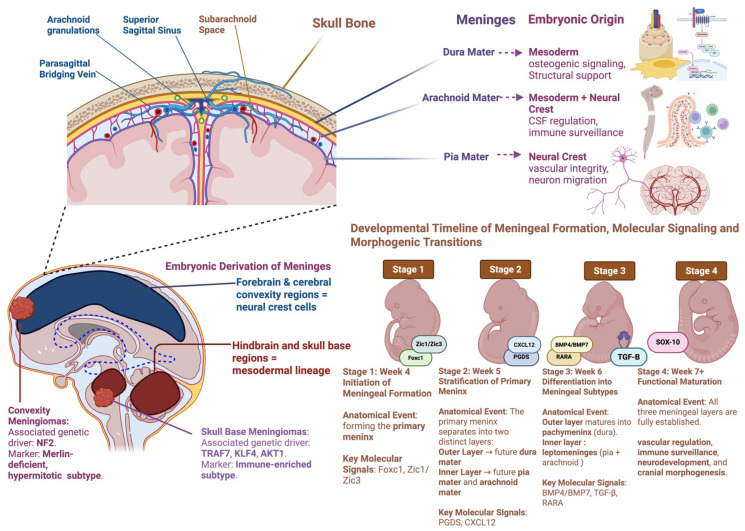
Embryonic Origins, Spatial Heterogeneity, and Developmental Timelines of the Meninges in Meningioma Tumorigenesis. This figure integrates anatomical, developmental, and molecular perspectives on meningeal embryogenesis and its relevance to meningioma biology. The upper panel illustrates the three meningeal layers: dura mater (mesoderm-derived), arachnoid mater (mesoderm + neural crest), and pia mater (neural crest-derived), and their respective functions in structural support, CSF regulation, immune surveillance, and neurovascular development. The bottom left panel maps embryonic derivation across brain regions, highlighting that forebrain and convexity meninges arise from neural crest cells. In contrast, the hindbrain and skull base meninges derive from mesoderm, paralleling the spatial and genetic divergence seen in NF2-driven convexity meningiomas and TRAF7/KLF4-driven skull base subtypes. The right panel presents a developmental timeline of meningeal formation from mesenchymal condensation (Week 4) through layer stratification (Week 5), differentiation (Week 6), and functional maturation (Week 7+), alongside key molecular regulators including FOXC1, ZIC1/3, CXCL12, BMP4/7, TGF-β, and RARA. This figure underscores the embryologic and molecular scaffolding that may underlie spatial heterogeneity and therapeutic vulnerability in meningioma. Created with BioRender.com.

The diverse and divergent roles of various genes and transcription factors in neural crest and mesodermal cells can also partially explain the limitations of current clinical studies [102,112]. For example, therapeutic mesenchymal stem cells (MSCs) showed no therapeutic efficacy for forebrain and skull disorders. MSCs are of mesodermal origin, which may explain why they are less efficient in neural-crest dominant regions (i.e., forebrain). Accordingly, the regenerative application of neural-crest-derived MSCs for forebrain disorders has been of particular interest to overcome these limitations [95,114,115]. Figure 2 recapitulates the embryonic origins, spatial heterogeneity, and developmental timelines of the meninges in meningioma tumorigenesis.

Moving forward, a previously unexplored area of research is the distinct differential genomic expression and potential cellular vulnerability in either neural crest or mesodermal cells. Further characterization of dominant neural crest and mesodermal brain regions may be the way to account for phenotype diversity and different genomic expressions. One promising and gold-standard strategy to assess cell-type-specific genomic expression is to test the accessibility of DNA structure. Specifically, the role of chromatin accessibility is a potential tool to unravel the molecular basis that drives aberrant biochemistry and regulatory mechanisms in meningeal development and associated disorders. Table 2 recapitulates the molecular and immunological features of the meningioma microenvironment.

## 4. Biological Drivers and Therapeutic Vulnerabilities

Given the substantial heterogeneity and unpredictability in dysregulation, amplification, proliferation, and mutational patterns in high-grade and recurrent meningiomas, identifying distinct genotypic features and phenotypic drivers with direct clinical associations is of substantial therapeutic significance. The last decade’s DNA methylation profiling, emergent transcriptomics, proteomics, and single-cell sequencing approaches have yielded three distinguished meningioma molecular profiles with distinct clinical outcomes [44,116,117,118].

Epigenomic studies more recently have shown that meningiomas can be broadly stratified into molecular subgroups with disparate clinical behaviors, immune contexts, and treatment sensitivities. Of these, DNA methylation-based profiling has demarcated subtypes with association to Merlin/*NF2* pathway status, immune enrichment with HLA and lymphatic signatures as predominant characteristics, and hypermitotic activity with such characteristics as overexpression of FOXM1 and silencing of *CDKN2A/B*. These molecular subgroups correlate with differential prognoses and highlight the potential of epigenetic classifiers to guide personalized treatment protocols and risk-adapted clinical surveillance [72,119,120,121,122].

The mechanistic consensus in the majority of contemporary molecular profiling studies on meningiomas is that cytostatic cell cycle inhibitors reduce meningioma proliferation and progression across cell cultures, organoids, patient-derived xenografts, and recurrent meningioma patients [72,123]. This is primarily due to two reasons. First, it is well characterized in the literature that hypermitotic and immune-enriched meningiomas have highly unfavorable clinical outcomes, substantial cell proliferation, and carry nearly total resistance to cytotoxic therapies due to misactivation of FOXM1 and loss of Merlin/*NF2*. Second, numerous studies have demonstrated that Merlin/*NF2-*intact meningiomas have the most favorable prognosis and are well-suited to existing therapeutics [29,123,124]. Merlin further shows this by regulating glucocorticoid receptor signaling pathways and is the leading inducer of meningioma apoptosis [124]. Table 3 recapitulates the current and emerging therapeutic strategies in meningiomas.

Substantial cell cycle proliferation characterizes both mechanisms underlying the above-described cell cycle misactivation and cytotoxic resistance in hypermitotic and immune-enriched meningiomas. Hence, cytostatic cell cycle inhibitor drugs, such as DNA intercalating agents that target patient-stratified methylation patterns in meningiomas, may serve as effective molecular-based therapeutics for high-grade and recurrent meningiomas [65,72,126]. Multiple studies have demonstrated the potential efficacy of the CDK4/6 cell cycle inhibitors palbociclib and abemaciclib in blocking the clonogenic growth of meningioma cells without disruption and modification of normal cell cycle behavior [72,126]. This particular therapeutic efficacy has been demonstrated in the context of the meningioma tumor microenvironment through the co-culturing of human induced pluripotent stem cell (iPSC) models of cerebral organoids with highly heterogeneous meningioma cells [72,127,128]. This is a particularly promising venture to address the therapeutic vulnerabilities of meningiomas for two reasons. First, the predominance of high intratumoral heterogeneity in their meningioma cells is a significant limitation in the patient-stratified therapeutic strategy for high-grade anaplastic and atypical meningioma patients. Second, high intratumoral heterogeneity is the primary reason for resistance to the current therapies and adverse clinical outcomes in high-grade and recurrent meningiomas. Hence, developing therapeutic models that can recapitulate the highly heterogeneous microenvironment of meningiomas will serve as the most promising approach to developing patient-specific treatment strategies and discovering target-based drugs in high-grade and recurrent meningiomas.

## 5. Advanced Thematic Considerations in Meningioma Spatial Oncogenesis and Targeting

### 5.1. Spatial and Lineage-Specific Origins of Anaplasia in Convexity Meningioma

Anaplastic meningiomas are most commonly found in the convexity, falx, and parasagittal sites, areas embryologically derived from neural crest–origin meninges. This spatial enrichment mirrors their genomic profile: frequent *NF2* inactivation, high-grade chromosomal instability, *CDKN2A/B* homozygous deletion, and 1p/14q co-loss [129,130,131]. Unlike their mesodermal skull base counterparts, convexity meningiomas have more common methylation-based epigenetic silencing and transcriptomic overrepresentation of cell cycle and chromatin remodeling pathways [132,133,134].

This suggests a working hypothesis: neural crest-derived meninges may have greater epigenetic plasticity and dedifferentiation susceptibility following *NF2* loss. Within these contexts, YAP/TAZ signaling, activated following *NF2* loss, is increasing cellular stemness and proliferation, particularly in neural crest–lineage tissues [43,135,136]. The transcriptional and chromatin landscape setting of neural crest-derived meninges is more permissive to reprogramming [74], which may be culprits in regional preference for anaplastic transformation.

Therapeutically, these data argue that *NF2/YAP* inhibition alone may be insufficient. Epigenetic regulator inhibition such as EZH2 inhibitors, BET bromodomain inhibitors, or demethylating agents, may be combined with YAP/TAZ inhibition to suppress dedifferentiation and malignant growth [136]. Spatially informed stratification by embryologic origin may be employed to identify high-risk patients for anaplastic transformation and implement early targeted intervention.

### 5.2. Targeting TRAF7: Challenges and Emerging Strategies

*TRAF7* mutations are found to be enriched in anterior skull base meningiomas, which are mesodermal in origin and tend to have non-*NF2* genomic patterns [43]. As an E3 ubiquitin ligase, *TRAF7* targets pivotal signaling pathways, such as NF-κB, JNK, and apoptotic pathways, without having a traditional enzymatic pocket, thereby rendering it undruggable by traditional small-molecule approaches. Its pleiotropic signaling role further contributes to the complexity of direct inhibition, with a higher likelihood of systemic toxicity [137].

To prevent this, the therapeutic focus is shifting to targeting downstream networks that are *TRAF7*-dependent. Aberrant NF-κB signaling, for example, provides a tractable axis upon which to repurpose NF-κB inhibitors in the pipeline [120,138,139]. Tumor mutants for *TRAF7* also have vulnerabilities in ER stress response or proteostasis pathways and rationale exists for the investigation of ER stress modulators or therapies aimed at proteostasis.

Synthetic lethality approaches are an emerging paradigm in oncology [140]. Synthetic lethality permits indirect targeting of non-druggable oncogenic drivers by employing cancer-specific genetic dependencies. Advances in CRISPR-based screening have disclosed such interactions in DNA repair, epigenetic, and stress-response pathways. We propose that *TRAF7*-mutant meningiomas possess synthetic lethal vulnerabilities in MAPK or ER stress signaling, which can be discovered by lineage-specific CRISCR screening in meningeal models. Furthermore, *TRAF7*-mutant meningiomas may possess compensatory dependencies on MEK/ERK or JNK pathways, which can be co-inhibited to induce preferential killing. In parallel, new PROTAC-based degraders [141], promise the most direct entry to targeting *TRAF7* itself, beyond ligandable sites.

Given the rarity and near mutual exclusivity of *TRAF7* mutations, future clinical trials can be enhanced through spatial–genomic stratification to heighten biomarker specificity, reduce intertumoral heterogeneity, and enable rational introduction of synthetic lethal or pathway-specific therapies for mesoderm-derived skull base meningiomas.

## 6. Conclusions

Meningioma biology is increasingly defined by the intersection of developmental lineage, epigenetic plasticity, and immune–tumor crosstalk. Spatial–genomic approaches identify that meninges of neural crest and mesodermal origin impart unique mutational and epigenetic programs that determine the behavior of tumors and therapeutic vulnerability. Recurrent and high-grade meningiomas are characterized by hypermitotic and immune-enriched states characterized by FOXM1 activation, loss of *CDKN2A/B*, and *NF2* disruption, leading to a context-dependent proliferative but tolerogenic microenvironment with M2-polarized macrophages and lymphocytic infiltration. Multi-omic profiling, DNA methylation classifiers, single-cell and spatial transcriptomics, now supports patient-stratified approaches that integrate embryologic origin and molecular subtype and immune microenvironment.

Future therapeutic approaches will be required to take advantage of this integrative biology. Epigenetic modifiers and CDK4/6 inhibitors are therapeutically appealing against hypermitotic and immune-potent tumors, and synthetic lethal approaches and targeting the *TRAF7* pathway potentially provide precision therapy to mesoderm-derived subtypes. The immunologic space, where epigenetic regulation of antigen presentation and macrophage polarization is the basis of recurrence, is also the site of parallel avenues for immune manipulation. Main messages: (1) Spatial–genomic architecture is developmental lineage-based in meningioma; (2) Immune and epigenetic programs control progression and resistance; (3) Multi-omic stratification underpins rational, patient-specific treatment; (4) Synthetic lethal and immune-based strategies are key future directions for recurrent and high-grade disease.

Given the challenges posed by the unique tumor microenvironment and molecular complexity of meningiomas, future studies should further deploy multi-omics including single-cell and spatial transcriptomics, proteogenomics, and metabolomics to refine diagnostic frameworks and therapeutic interventions. Ultimately, a molecularly guided patient-stratified approach could be central to optimizing outcomes for patients with aggressive and recurrent meningiomas.

## Figures and Tables

**Table 1:** Key Genetic and Epigenetic Alterations in Meningioma.

Gene/Pathway	Molecular Role	Associated Meningioma Subtype	Implications
*NF2* (Merlin)	Tumor suppressor	Merlin-intact	Loss of function leads to dysregulated PI3K/mTOR and Hippo signaling, promoting tumorigenesis
*TRAF7*	E3 ubiquitin ligase	Skull base meningiomas	Frequently mutated in non-*NF2* meningiomas, influencing MAPK signaling
*CDKN2A/B*	Cell cycle regulators	Hypermitotic meningiomas	Frequent homozygous deletions drive high proliferation and poor prognosis
*TERT* Promoter	Telomerase activation	High-grade meningiomas	Associated with aggressive tumor behavior and recurrence
Epigenetic Dysregulation	DNA methylation & histone modifications	All subtypes (esp. hypermitotic)	Drives heterogeneity, affects the response to therapy
*PIK3CA*	Catalytic sub-unit of PI3 K	Non-*NF2* skull base meningothelial meningiomas	Mutations drive constitutively active PI3K signaling, enriched for hormone receptor positive meningiomas
*SMO*	G-PCR in Hedgehog Signaling	Non-*NF2* skull base meningiomas	Activating mutations trigger GLI dependent tumor growth
*KLF4*	TF in epithelial differentiation	Secretory meningiomas	K409Q mutation causes mucin rich secretory phenotype
*AKT1*	PI3K downstream kinase	Meningothelial meningiomas	Constitutively active PI3K signaling, moderate recurrence risk
CNV 1p/22q	Tumor suppressor loci CNV	High grade and aggressive low grade meningiomas	Loss of tumor suppressors, increase in molecular grade

**Table 2:** Molecular and Immune Features of Meningioma Microenvironment.

Tumor Microenvironment Component	Function in Meningioma	Therapeutic Implications
Immune Cells (TAMs, Tregs, MDSCs)	Immunosuppressive, promote tumor progression	Potential for immune checkpoint inhibitors
Extracellular Matrix (ECM)	Supports tumor adhesion, invasion	Targeting ECM remodeling (MMP inhibitors)
Hypoxia & Angiogenesis	VEGF-mediated neovascularization	Anti-angiogenic therapies (Bevacizumab)
Cytokines (IL-6, IL-10, TGF-β)	Immune evasion, therapy resistance	Targeted cytokine inhibition strategies

**Table 3:** Current and Emerging Therapeutic Strategies for Meningioma.

Therapy Type	Target/Mechanism	Indications	Current Status
Surgical Resection	Gross total resection	Grade I–III meningiomas	Gold standard, but limited by tumor location
Radiation Therapy	Fractionated, SRS	Incompletely resected or recurrent tumors	Standard adjunct for high-grade or recurrent cases
CDK4/6 Inhibitors (Palbociclib, Abemaciclib)	Cell cycle blockade	Hypermitotic meningiomas	Preclinical success, clinical trials underway
Somatostatin Analogues (Octreotide, Pasireotide)	SST2 receptor inhibition	*NF2*-null and *TRAF7*-mutated meningiomas	Some efficacy in non-*NF2* meningiomas
Anti-VEGF Therapy (Bevacizumab)	Angiogenesis inhibition	High-grade and recurrent meningiomas	Mixed clinical trial results
Organoid & iPSC-based Models	Precision medicine approach	All meningioma subtypes	Emerging tool for patient-specific therapies
*SMO* Inhibitors (Vismodegib)	Hh signaling inhibitor	Progressive meningiomas with SMO mutation	Phase II clinical trials underway [125]
PI3K/AKT axis inhibitors (Capivasertib, Alpeslisib)	PI3Ka, *AKT1* or mTORC1/2 blockade	Progressive meningiomas with mutations in PI3Ka or *AKT1* and related pathways	Phase I clinical trials of Alpeslisib underway, Phase II clinical trials of Capivasertib underway
FAK inhibitors (GSK2256098)	Focal adhesion kinase inhibition exploiting loss of NF2/merlin	*NF2*-null grade I–III meningiomas	Mixed early clinical trial results, phase II clinical trials underway
Hippo/YAP-TEAD inhibitors (IK-930, VT-3989)	Shuts down YAP/TAZ transcription	*NF2*-null or *YAP*-activated meningiomas	Ongoing and recruiting phase I clinical trials. VT-3989 resulted in partial response of 9 meningioma patients [68].

## Data Availability

No new data were created or analyzed in this study. Data sharing is not applicable to this article.
